# Socioeconomic factors and outcome after repair and reconstruction of digital and major nerve trunk injuries in the upper limb

**DOI:** 10.1038/s41598-024-57757-w

**Published:** 2024-03-27

**Authors:** Drifa Frostadottir, Raquel Perez, Lars B. Dahlin

**Affiliations:** 1 Department of Translational Medicine-Hand Surgery, Lund University, Skåne University Hospital, Jan Waldenströms Gata 5, 205 02 Malmö, Sweden; 2 Department of Hand Surgery, Skåne University Hospital, Malmö, Sweden; 3 Unit for Social Epidemiology, Department of Clinical Sciences (Malmö), Lund University, Malmö, Sweden; 4 Department of Biomedical and Clinical Sciences, Linköping University, Linköping, Sweden

**Keywords:** Digital nerve injury, Major nerve trunk injury, Peripheral nerve injury, Socioeconomic factors, Surgery outcomes, Peripheral nervous system, Signs and symptoms, Socioeconomic scenarios, Health care

## Abstract

Peripheral nerve injuries in the upper limb can lead to substantial disability and pain. We aimed to assess how socioeconomic factors affect outcomes after repaired or reconstructed digital or major nerve trunk injuries in the upper limb. We identified 670 individuals, who underwent surgical nerve repair or reconstruction using sensory nerve autografts, in the Swedish National Quality Registry for Hand Surgery 2010–2018. Socioeconomic factors, including education, cohabitation, type of work, sick leave, immigrant status and income, were gathered from the Swedish statistical agency (www.scb.se) and National Diabetes Register (NDR). We calculated prevalence ratios (PR) to assess the relationship between socioeconomic factors and surgical outcomes for the nerve injuries. Individuals with a major nerve trunk injury had higher QuickDASH scores and lower income compared to those with digital nerve injury. Individuals with immigration background (adjusted PR = 2.0, 95% CI 1.2–3.2), history of > 4 weeks of sick leave the year before surgery (adjusted PR = 1.8, 95% CI 1.1–3.1), or education level below tertiary (adjusted PR = 2.8, 95% CI 1.7–4.7) had significantly higher QuickDASH scores. Recognizing impact of non-biological factors, including immigration, prior sick leave, and education level, on outcome after nerve surgery is crucial for improving prognosis in socioeconomically deprived individuals.

## Introduction

Peripheral nerve injuries can result in significant and enduring disability and pain. These disabilities can severely limit daily activities, work participation, and social interactions, potentially leading to reduced self-esteem^1,2^.

Nerve injuries often affect individuals in their working years, incurring substantial personal and societal costs^3–5^. Digital nerves, more commonly affecting men^6,7^, are more frequently injured than the median and ulnar nerves^8,9^. A severe pain condition after a nerve injury may necessitate not only surgical intervention but also pharmacological treatment, carrying the risk of side effects and potential drug dependence, further burdening individuals, and society^10,11^. Disabling and persistent pain can affect the individual’s physical and mental health with reduced quality of life, depression, and suicide risk^12,13^. Therefore, understanding the factors that influence outcomes, pain risk, and return to work remains crucial.

Socioeconomic status is the social standing or class of an individual or group, often defined as a combination of education, income, and occupation, and may be related to outcome of surgery. Previous studies have shown that certain socioeconomic factors, such as lower education level, low income, immigrant status and frequent sick leave, are associated with more symptoms both before and after surgery for carpal tunnel syndrome (CTS)^14,15^. Yet, no studies have described the association between socioeconomic factors and outcome after repaired or reconstructed nerve injuries in the upper limb. Our aim was to study the impact of socioeconomic factors on outcome after repaired or reconstructed nerve injuries in the upper limb, including both digital nerve and major nerve trunk injuries.

## Methods

### Study design, participants, and data sources

In this retrospective study, we focused on individuals aged 20–64 years who underwent surgical nerve repair or reconstruction using nerve autografts for single digital or major nerve trunk injuries (e.g., median, ulnar, or radial nerve injuries at the wrist or forearm) between 2010 and 2018. We identified individuals from the Swedish National Quality Registry for Hand Surgery (HAKIR)^16^ using ICD-10 [International Statistical Classification of Diseases and Related Health Problems^17^] diagnosis codes S644, S643, S640, S641, S642 S540, S541 and S542 and surgical procedure codes (KKÅ97) ACB29. ACB21, ACB22, ACB23, ACC22, ACC23, and ZZK00.

Patient-reported outcomes related to peripheral nerve injuries in the upper limb were collected through postal or online methods and registered in HAKIR^16^. This included the validated Swedish version of the QuickDASH questionnaire, scored from 0 to 100, where higher scores indicate greater disability^18^. Socioeconomic information, such as education level, marital status, employment status, sick leave, immigrant status, and income, was obtained from the Swedish statistical agency (www.scb.se). We used QuickDASH scores of 40 or higher for individuals with digital and major nerve trunk injuries postoperatively to investigate the relationship between socioeconomic status and outcome. An individual with a QuickDASH score below 15 has previously been described as having “no problem,” whereas a score falling within the rage of 40–69 indicated “significant difficulties and inability to work”^19^. Additionally, we linked data from the Swedish National Diabetes Register (NDR; http://www.ndr.se), to the HAKIR database to determine the diabetic status of individuals diagnosed with type 1 or type 2 diabetes above the age of 20 years. Informed consent was obtained from each individual before inclusion in the registries. Exclusion criteria included surgery involving nerve biopsy, combined injuries to both digital nerves and major nerve trunks, surgery for multiple nerve injuries, and age < 20 and > 64 years due to limited availability of socioeconomic data for these age groups from Swedish statistical agency. Data was available from 2009 to 2017.

Marital status included single, married/registered partner, divorced, or widowed. We documented marital status in the year preceding surgery and grouped individuals into those cohabiting or not.

Data on employment or unemployment were collected from the year before the individual's surgery and categorized into manual and non-manual employment types.

Education level was obtained from the year before operation and divided into three groups based on the International Standard Classification of Education (ISCED): primary (ISCED 0, 1, and 2, representing ≤ 9 years of education or compulsory school), upper secondary (ISCED 3, representing 9–12 years of education), and tertiary (ISCED 4, 5, and 6, representing > 12 years of education).

Data regarding earned income was available from 2009 to 2017. Earned income was based on income the year before injury, a binned variable was created using the 25th, 50th and 75th percentiles compared to the Swedish population by sex and year, based on data from the Swedish statistics Agency (SCB).

Sick leave was calculated as net days (one day with 100% sick leave counts as one net day, one day with 50% sick leave counts as 0.5 net days etc.). In Sweden, the social security system involves the employer paying for sick leave during the first 14 consecutive days for individuals employed for at least 6 months. A binned variable was created based on patient with registered sick leave of 14 consecutive days or more, amounting to a total of at least 4 weeks the year before injury.

Immigration background was categorized as native for individuals born in Sweden and immigrant for those born outside of Sweden.

Information on diabetes included type 1 and 2 diabetes.

### Statistics

Given the relatively high prevalence of outcomes, we used prevalence ratios (PRs) rather than odds ratios in our analysis^20^. We employed a Cox proportional hazards regression model with a constant follow-up time of 1. We developed eleven consecutive regression models. Model 1 included only age and used univariate regression (unadjusted), while model 2 was adjusted for socioeconomic, demographic, and health variables (i.e., age, sex, income, immigration status, sick leave, employment type, education, type of injury, and diabetes).

To assess the discriminatory accuracy (DA) of each model, we calculated the area under the receiver operating characteristic curve (AUC) and its 95% confidence intervals (CI). The AUC values ranged from 0.5 (indicating no predictive accuracy)^21^ to 1 (representing perfect discrimination). We classified the (DA) based on criteria proposed by Hosmer and Lemeshow^22^ as absent or very weak (AUC = 0.5–0.6), poor (AUC > 0.6– ≤ 0.7), acceptable (AUC > 0.7– ≤ 0.8), excellent (AUC > 0.8–0.9), or outstanding (AUC > 0.9).

Data are presented as mean ± SD, prevalence ratio (PR) with 95% confidence interval (CI), or numbers (%). We used t-tests for continuous variables (age and QuickDASH score) and the Chi-Square test for categorical variables. ANOVA analysis with Bonferroni comparison and Tukey test was applied to assess mean differences among more than two groups.

For the expanded Cox proportional hazards regression models, drop out analysis and significant associations of baseline characteristics and socioeconomic factors; see Supplemental Digital Content.

### Ethical approval and consent to participate

This study was approved by the Regional Ethical Review Board in Stockholm, Sweden, and the national Ethical Review Board (2017/2023: 31; 2018/1106-32; 2019-00880; 2021-0418 and 2021-00902). The research was conducted in accordance with the principles of the Helsinki Declaration. Individuals provided informed written consent before inclusion in HAKIR and NDR.

## Results

### Included individuals

**Figure 1:**
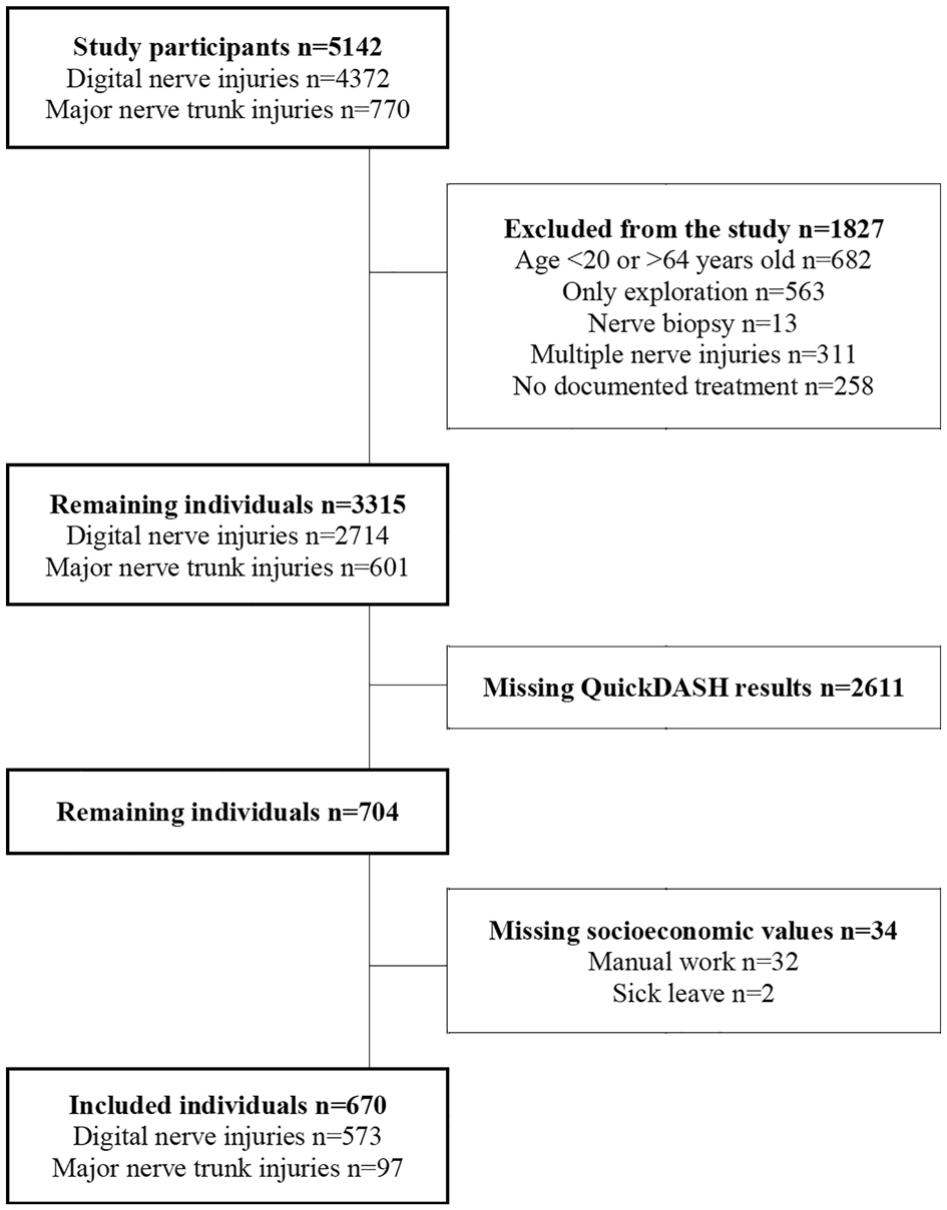
Study patient flow chart showing included individuals in the study.

During the study period, 5142 individuals with digital nerve and major nerve trunk injuries (4372 and 770 individuals, respectively) were identified in the national quality registry for hand surgery (HAKIR; hakir.se; Fig. 1). Of these, 682 individuals were excluded due to age (< 20 to > 64 years). Among the remaining individuals, 563 underwent exploration only, 13 had nerve biopsies for nerve tumour, 311 had multiple upper extremity nerve injuries (including ICD-10 code S64.7, covering both digital nerves and major nerve trunks), and 258 had no documented treatment for their nerve injuries, leading to their exclusion. This left 3315 eligible individuals: 2714 with digital nerve injuries and 601 with major nerve trunk injuries, treated with direct nerve repair or nerve grafting, with or without concomitant arm/hand injuries. Of these individuals, 704 (21%) responded to the QuickDASH at 12 months postoperatively. There were 34 individuals with missing socioeconomic data (32 for type of work and 2 for sick leave the year before the operation). In the end, the study included 670 individuals (573 with digital nerve injuries and 97 with major nerve trunk injuries) (Fig. 1).

### Baseline characteristics

Among the individuals included, 424/670 (63%) were men, and 246/670 (37%) were women. There was no significant age difference between the two nerve injury groups: those with digital nerve injuries had a mean age of 41 ± SD 13 years, while those with major nerve trunk injuries had a mean age of 39 ± SD 13 years (p = 0.078). Individuals with digital nerve injuries had a higher income than those with major nerve trunk injuries (p = 0.031). There were no significant differences in sex (p = 0.083), immigrant status (p = 0.120), sick leave (p = 0.502), education level (p = 0.839), type of work (p = 0.114), cohabiting status (p = 0.082), or pre-existing diabetes (p = 0.820) between individuals with digital nerve injuries and those with major nerve trunk injuries (Table 1). Pre-existing diabetes was diagnosed in 16/670 (2.4%) individuals, with 6 having type 1 diabetes and 10 having type 2 diabetes.

**Table 1:** Baseline characteristics and socioeconomic factors of individuals treated with repair or reconstruction of a digital nerve or major nerve trunk injury in the upper extremity.

Baseline characteristics				
		Digital nerve injury n = 573 (86)	Major nerve trunk injury n = 97 (14)	p value
QuickDASH score				
	0–20	345 (60)	32 (33)	
	21–40	154 (27)	38 (39)	
	41–60	56 (10)	14 (14)	
	61–80	16 (3)	9 (9)	
	81–100	2 (0.5)	4 (4)	
Mean QuickDASH score		20 ± SD 17	31 ± SD 22	** < 0.0001**
Diabetes				
	No	559 (98)	95 (98)	0.82
	Yes	14 (2)	2 (2)	
Age (years)				
	20–34	190 (33)	43 (44)	
	34–54	266 (46)	37 (38)	
	55–64	117 (20)	17 (18)	
Mean age		41 ± SD 13	39 ± SD 13	0.078
Sex				
	Men	355 (62)	69 (71)	0.083
	Women	218 (38)	28 (29)	
Immigrant status				
	Immigrant	63 (11)	16 (17)	0.120
	Native	510 (89)	81 (84)	
Sick leave^a^				
	No	500 (87)	87 (90)	0.502
	Yes	73 (13)	10 (10)	
Education level				
	Primary	79 (14)	12 (12)	0.839
	Upper secondary	111 (19)	21 (22)	
	Tertiary	383 (67)	64 (66)	
Manual work				
	No	250 (44)	34 (35)	0.114
	Yes	323 (56)	63 (65)	
Cohabiting				
	No	282 (49)	57 (59)	0.082
	Yes	291 (51)	40 (41)	
Income level				
	Low	133 (23)	27 (28)	**0.031**
	Middle-low	124 (22)	24 (25)	
	Middle-high	158 (28)	33 (34)	
	High	158 (28)	13 (13)	

Data presented as n (%).

Bold values indicate p value < 0.05.

^a^ ≥ 4 weeks the year before injury.

The model was adjusted for concomitant injuries, revealing no confounding, or modifying associations between the primary socioeconomic predictors and the outcome. A dropout analysis found no differences in baseline characteristics or socioeconomic factors between included and excluded individuals (Supplemental Digital Content: S1).

### Nerve injury and socioeconomic factors

A significant difference emerged between individuals with digital nerve injuries and major nerve trunk injuries. Those with major nerve trunk injuries scored 11 points higher on the QuickDASH questionnaire (p < 0.0001) (Table 1) and had a higher risk of scores exceeding 40 at 12 months (unadjusted PR = 2.2; 95% CI 1.4–3.3 and adjusted PR = 1.9; 95% CI 1.2–3.0) (Table 2).

**Table 2:** Cox proportional hazard regression analysis (time constant) showing unadjusted and adjusted outcome of individuals with a score of 40 or higher on QuickDASH questionnaire.

Cox proportional hazard regression analysis			
		Unadjusted PR	Adjusted PR
Nerve injury			
	Digital nerve injury	Reference	Reference
	Major nerve trunk injury	**2.2 (1.4–3.3)**	**1.9 (1.2–3.0)**
Diabetes			
	No	Reference	Reference
	Yes	1.7 (0.6–4.6)	1.7 (0.6–4.7)
Age (years)			
	20–34	1.3 (0.8–2.3)	1.4 (0.7–2.6)
	34–54	1.0 (0.6–1.8)	1.3 (0.7–2.2)
	55–64	Reference	Reference
Sex			
	Men	Reference	Reference
	Women	1.1 (0.7–1.6)	1.3 (0.8–1.9)
Immigrant status			
	Immigrant	**2.2 (1.4–3.5)**	**2.0 (1.2–3.2)**
	Native	Reference	Reference
Sick leave^a^	No	Reference	Reference
	Yes	**1.6 (1.0–2.7)**	**1.8 (1.1–3.1)**
Education level			
	Primary	**2.9 (1.8–4.7)**	**2.8 (1.7–4.7)**
	Upper secondary	**1.9 (1.2–3.0)**	**2.1 (1.2–3.6)**
	Tertiary	Reference	Reference
Manual work			
	No	Reference	Reference
	Yes	**1.8 (1.2–2.8)**	1.3 (0.8–2.0)
Cohabiting			
	No	**1.6 (1.1–2.4)**	1.4 (0.9–2.3)
	Yes	Reference	Reference
Income			
	Low	**2.9 (1.6–5.4)**	1.8 (0.9–3.4)
	Middle-low	**1.9 (1.0–3.7)**	1.3 (0.6–2.5)
	Middle-high	1.7 (0.9–3.2)	1.2 (0.6–2.3)
	High	Reference	Reference

Statistically significant associations presented in bold. Data presented as PR = prevalence ratio, 95% CI = confidence interval. (For the expanded version showing all 11 models; see Supplemental Digital Content: S2).

^a^ ≥ 4 weeks the year before injury.

**Figure 2:**
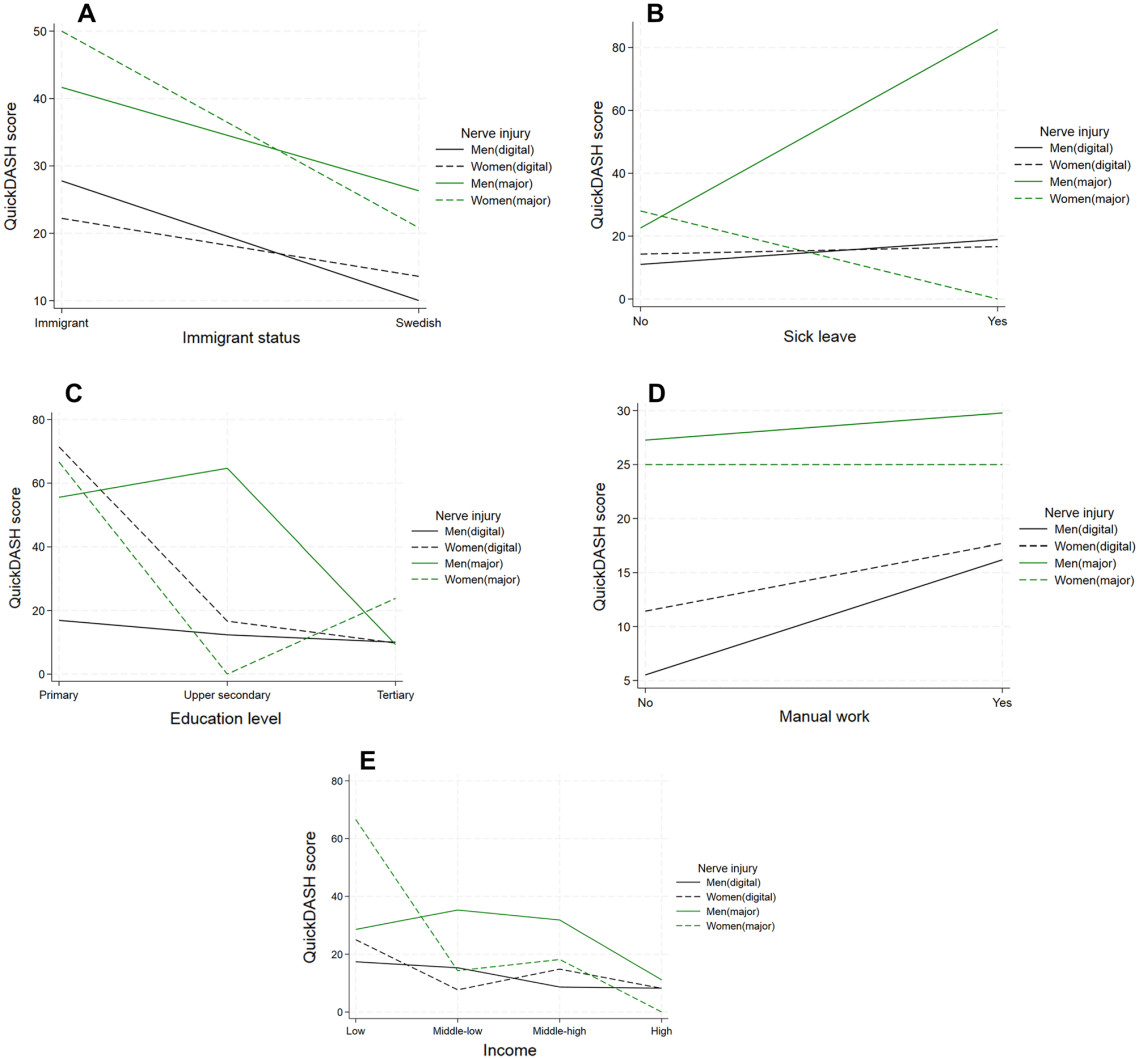
QuickDASH score. Absolute risk of worse QuickDASH score (< 40), presented for men and women with a digital nerve injury or a major nerve trunk injury respectively. (**A**) Swedish born and immigrants. (**B**) Having had 4 weeks or more of sick leave in the year prior injury. (**C**) With primary, upper secondary and tertiary education. (**D**) With manual and non-manual work. (**E**) With low, middle-low, middle-high, and high income.

Immigrants to Sweden exhibited a notably higher risk for worse QuickDASH score (> 40) compared to those born in Sweden (unadjusted PR = 2.2, 95% CI 1.4–3.5 and adjusted PR = 2.0, 95% CI 1.2–3.2) (Table 2), particularly among women with major nerve trunk injuries (Fig. 2A).

Individuals with  ≥ 4 weeks of sick leave the year before surgery had significantly higher risk for QuickDASH scores > 40 (unadjusted PR = 1.6 95% CI 1.0–2.7 and adjusted PR = 1.8, 95% CI 1.1–3.1) (Table 2). This was most prominent in men with a major nerve trunk injury, with a reverse trend observed for women with a major nerve trunk injury (Fig. 2B).

Lower than tertiary education was associated with risk for worse QuickDASH scores (> 40) (unadjusted PR = 2.9, 95% CI 1.8–4.7 and adjusted PR = 2.8, 95% CI 1.7–4.7) (Table 2). This was most pronounced among men with a major nerve trunk injury (Fig. 2C).

Manual work was linked to a risk of higher QuickDASH scores (> 40) compared to non-manual work (unadjusted PR = 1.8; 95% CI 1.2–2.8) (Table 2). This was most pronounced among men with a digital nerve injury (Fig. 2D).

Lower income levels were associated with risk for worse QuickDASH scores (> 40) only before adjustment (unadjusted PR = 2.9; 95% CI 1.6–5.4) for those with low income compared to individuals with high income (Table 2). Notably, women with major nerve trunk injuries and low income had higher QuickDASH scores compared to men with similar income and major nerve trunk injuries (Fig. 2E).

When adjusted, the PR for manual work, cohabiting status, and income did not reach significance (Table 2).

The DA of the complete AUC model reached a value of 75% (Supplemental Digital Content: S2).

## Discussion

In this population-based study, we assessed socioeconomic status and its impact on QuickDASH scores after digital or major nerve trunk injuries. A major nerve trunk injury was associated with a risk for higher QuickDASH scores (> 40), with clinically relevant differences of 11 points^23^, signifying worse outcomes compared to a digital nerve injury. This difference persisted even after adjusting for age, sex, immigration, sick leave, education, manual work, cohabitation, income, and diabetes status. These findings align with prior research, where QuickDASH scores for surgically treated digital nerve injuries were generally lower (mean score 7–9)^24,25^ than those for individuals with a surgically treated major nerve trunk injury (mean score 29–31)^26^. Digital nerve injuries primarily affect finger sensation and fine motor skills, whereas major nerve trunk injuries impact both arm and hand motor function and sensation^27,28^, and both types of nerve injuries have a risk of residual pain problems, despite surgery. Consequently, major nerve trunk injuries typically entail greater functional limitations and rehabilitation needs, explaining the higher QuickDASH scores overall.

Immigrants to Sweden exhibited significantly higher QuickDASH scores than native-born individuals. This trend was consistent with nerve entrapment disorders, where immigrant women with surgically treated carpal tunnel syndrome (CTS) have 6 points higher postoperative QuickDASH scores at 12 months^15^. A similar pattern was observed in our study, particularly among immigrant women with a major nerve trunk injury. Studies on total hip replacement (THR) also indicated worse outcomes for immigrants at 12 months post-surgery, with dissatisfaction often attributed to inadequate pre-and postoperative information, a factor shown to reduce postoperative satisfaction^29^. Immigrant populations may face various challenges impacting their healthcare outcomes. Language, cultural barriers as well as health literacy disparities can hinder effective communication with healthcare providers, while limited access to interpretation services and culturally sensitive issues exacerbates these cases^30^. Thus, a biopsychosocial approach is recommended when treating individuals with nerve injuries^31^.

Individuals with ≥ 4 weeks of sick leave in the year before surgery had risk for higher QuickDASH scores (> 40). This trend is also noted in nerve entrapment disorders, where increased sick leave predicted worse postoperative QuickDASH scores at 12 months for CTS and ulnar nerve entrapment (UNE) patients^32^. A noticeable correlation emerged between higher QuickDASH scores and being a man with a major nerve trunk injury. These individuals typically had lower than tertiary education, were engaged in manual work, and had taken ≥ 4 weeks of sick leave the year before the injury. Lower education levels have a stronger impact on men’s health and mortality rates than on women’s^33,34^. Educational disparities can lead to more significant bodily pain, partly attributed to health literacy^35^. Previous research on manual laborers also reported worse motor recovery and lower return-to-work rates compared to office workers, with a median QuickDASH score at 12 months of 34 vs. 27, respectively^26^. Psychosocial factors, including depression, coping strategies, and anxiety, can influence outcome, like pain level, patient satisfaction, and disability^36^. The observed disparities in individuals with CTS are unrelated to psychological health or an increased risk of using painkillers, such as opioids^10,11^. Whether men with a major nerve trunk injury have poorer psychological health and higher risk of psychotropic or analgesic drug use is unknown.

Low-income levels were associated with risk for worse QuickDASH scores (> 40) before adjustment, especially among women with a major nerve trunk injury. Low income’s influence on QuickDASH scores is well-documented in the general population^37^. This association may stem from various factors, such as limited access to healthcare services, resulting in delayed diagnosis or treatment, physically demanding occupations, psychosocial stressors arising from socioeconomic deprivation, and lifestyle choices, like diet and exercise habits. However, additional research is required to validate these associations.

Although the number of individuals with diabetes was low (16/670; 2.4%), it aligned with diabetes prevalence in the general Swedish population^38^. Despite a higher prevalence of diabetes (around 12%) in population studies involving nerve entrapment disorders, like carpal tunnel syndrome and ulnar nerve compression, individuals with diabetes do not appear to have an elevated risk of upper limb nerve injury or worse outcomes after repair/reconstruction based on experimental studies^39,40^. This may be attributed to advancements in blood glucose monitoring, and other precautions concerning treatment of diabetes.

This study analysed 670 individuals treated for upper limb nerve injuries at seven university hospitals in Sweden over 8 years. Our results yielded the DA of the complete AUC model with a value of 75%, which is notably high supporting the reliability of our results. During this period, advancements in aftercare, including sensory training and cold sensitivity management^41,42^, may have improved outcomes. However, a closer examination of the data did not reveal any changes in the scoring trend over the years.

To our knowledge, this is the first study to highlight socioeconomic factors' impact on surgery outcomes for digital and major nerve trunk injuries in the upper limb. Our results highlight significant social disparities in individuals undergoing surgery for digital and major nerve trunk injuries, emphasizing the need for tailored individual treatment. Prolonged postoperative pain can lead to fear, helplessness, and demoralization, hindering patient engagement in recovery and satisfaction^43^. There is evidence to suggest that advanced pre-operative planning with expectation management can be helpful in improving surgical outcomes, including postoperative pain, improve coping with outcomes, and enhance patient satisfaction across various socioeconomic groups. Studies have shown that clear communication, realistic expectations, and involvement in decision-making can lead to better adherence to treatment plans and improved post-operative recovery^44^. Tailored treatment approaches may involve providing access to resources, such as financial assistance and transportation services, along with cultural competency training for healthcare providers, implementing multidisciplinary care teams, and offering health education programs. These measures could help addressing disparities related to socioeconomic status.

### Limitations

Apart from diabetes, our study did not account for medical comorbidities, smoking habits or tobacco use, the latter of which is higher among those with low socioeconomic status and a suggested risk factors for worse outcome after nerve surgery^45^. Furthermore, it did not include detailed information regarding specific surgical interventions or the type and duration of physiotherapy, known to influence the postoperative outcome^46^. Additionally, the relationship between immigration status and health outcome, including QuickDASH scores, varies widely in the literature, which can be influenced by a multitude of factors, including socioeconomic status, differences in access to healthcare in each country, cultural factors, such as health behaviors as well as language barriers. Therefore, these findings should be interpreted with caution.

## Conclusion

Poorer outcomes are common after major nerve trunk injuries compared to digital nerve injuries. Immigrants, those with extended sick leave, and individuals with lower education levels face a heightened risk of unfavourable QuickDASH scores (> 40). Emphasizing non-biological factors in clinical practice is crucial for improving outcomes in socioeconomically disadvantaged patients undergoing nerve surgery; thus, a biopsychosocial perspective is recommended in management of nerve injuries.

### Supplementary Information

Supplementary Tables.

## Data Availability

The datasets generated and/or analyzed during the current study are not publicly available. Public access to data is restricted by the Swedish Authorities (Public Access to Information and Secrecy Act; https://www.government.se/information-material/2009/09/public-access-to-information-and-secrecy-act/), but data can be available for researchers from the corresponding author after a special review that includes approval of the research project by both an Ethics Committee at the national level (www.etikprovningsmyndigheten.se) and the authorities’ data safety committees (such as “KVB-decision”).
