# Supplementary material for: Socioeconomic factors and outcome after repair and reconstruction of digital and major nerve trunk injuries in the upper limb
Source: Sci Rep. 2024 Mar 27;14:7242. doi: 10.1038/s41598-024-57757-w (PMC10973527; doi:10.1038/s41598-024-57757-w)
Supplement: Supplementary file 1 — Supplementary Tables. [file 41598_2024_57757_MOESM1_ESM.docx]

### Supplementary table 1 (S1)

| **Patient characteristics** | | | |
| --- | --- | --- | --- |
|  |  | Missing (outcome) | |
|  |  | No | Yes |
|  |  | 749 (23) | 2566 (77) |
| Age (years) |  |  |  |
|  | 20-34 | 261 (35) | 1229 (48) |
|  | 34-54 | 340(45) | 1026 (40) |
|  | 55-64 | 148 (20) | 311 (12) |
| Sex |  |  |  |
|  | Men | 474 (63) | 1781 (69) |
|  | Women | 275 (37) | 785 (31) |
| Immigration |  |  |  |
|  | Immigrants | 101 (13) | 605 (24) |
|  | Native | 648 (87) | 1958 (76) |
| Sick leave |  |  |  |
|  | No | 650 (92) | 2158 (91) |
|  | Yes | 58 (8) | 221 (9) |
| Education level |  |  |  |
|  | Primary | 109 (15) | 459 (18) |
|  | Upper secondary | 144 (19) | 517 (20) |
|  | Tertiary | 495 (66) | 1547 (60) |
| Manual work |  |  |  |
|  | No | 308 (43) | 876 (39) |
|  | Yes | 406 (57) | 1394 (61) |
| Cohabiting |  |  |  |
|  | No | 379 (51) | 1492 (58) |
|  | Yes | 370 (49) | 1069 (42) |
| Income |  |  |  |
|  | Low | 199 (27) | 990 (39) |
|  | Middle-low | 157 (21) | 549 (21) |
|  | Middle-high | 203 (27) | 521 (20) |
|  | High | 190 (25) | 506 (20) |
| Nerve injury | Digital nerve | 634 (85) | 2080 (81) |
|  | Major nerve trunk | 115 (15) | 486 (19) |
| Diabetes |  |  |  |
|  | No | 732 (98) | 2520 (98) |
|  | Yes | 17 (2) | 43 (2) |

Dropout analysis for the excluded individuals baseline characteristics and socioeconomic factors. Data presented as n (%).

### Supplementary table 2 (S2)

| **Cox proportional hazard regression model** | | | | | | | | | | | | |
| --- | --- | --- | --- | --- | --- | --- | --- | --- | --- | --- | --- | --- |
|  |  | Model 1 | Model 2 | Model 3 | Model 4 | Model 5 | Model 6 | Model 7 | Model 8 | Model 9 | Model 10 | Model 11 |
| Nerve injury | Digital | Reference |  |  |  |  |  |  |  |  |  | Reference |
|  | Major | 2.2(1.4-3.3) |  |  |  |  |  |  |  |  |  | 1.9(1.2-3.0) |
| Age (years) | 20-34 |  | 1.3(0.8-2.3) |  |  |  |  |  |  |  |  | 1.4(0.7-2.6) |
|  | 35-54 |  | 1.0(0.6-1.8) |  |  |  |  |  |  |  |  | 1.3(0.7-2.2) |
|  | 55-64 |  | Reference |  |  |  |  |  |  |  |  | Reference |
| Sex | Men |  |  | Reference |  |  |  |  |  |  |  | Reference |
|  | Women |  |  | 1.1(0.7-1.6) |  |  |  |  |  |  |  | 1.3(0.8-1.9) |
| Immigration | Immigrants |  |  |  | 2.2(1.4-3.5) |  |  |  |  |  |  | 2.0(1.2-3.2) |
|  | Native |  |  |  | Reference |  |  |  |  |  |  | Reference |
| Sick leave | No |  |  |  |  | Reference |  |  |  |  |  | Reference |
|  | Yes |  |  |  |  | 1.6(1.0-2.7) |  |  |  |  |  | 1.8(1.1-3.1) |
| Education | Primary |  |  |  |  |  | 2.9(1.8-4.7) |  |  |  |  | 2.8(1.7-4.7) |
|  | Upper secondary |  |  |  |  |  | 1.9(1.2-3.0) |  |  |  |  | 2.1(1.2-3.6) |
|  | Tertiary |  |  |  |  |  | Reference |  |  |  |  | Reference |
| Manual work | No |  |  |  |  |  |  | Reference |  |  |  | Reference |
|  | Yes |  |  |  |  |  |  | 1.8(1.2-2.8) |  |  |  | 1.3(0.8-2.0) |
| Cohabiting | No |  |  |  |  |  |  |  | 1.6(1.1-2.4) |  |  | 1.4 (0.9-2.3) |
|  | Yes |  |  |  |  |  |  |  | Reference |  |  | Reference |
| Income | Low |  |  |  |  |  |  |  |  | 2.9(1.6-5.4) |  | 1.8(0.9-3.4) |
|  | Middle-low |  |  |  |  |  |  |  |  | 1.9(1.0-3.7) |  | 1.3(0.6-2.5) |
|  | Middle-high |  |  |  |  |  |  |  |  | 1.7(0.9-3.2) |  | 1.2(0.6-2.3) |
|  | High |  |  |  |  |  |  |  |  | Reference |  | Reference |
| Diabetes | No |  |  |  |  |  |  |  |  |  | Reference | Reference |
|  | Yes |  |  |  |  |  |  |  |  |  | 1.7(0.6-4.6) | 1.7(0.6-4.7) |
| ROC |  | 0,5722 | 0,5358 | 0,5112 | 0,5647 | 0,5378 | 0,6304 | 0,5805 | 0,5694 | 0,6173 | 0,5093 | 0,7480 |

An expanded Cox proportional hazards regression model for individuals with a score of 40 or higher on QuickDASH questionnaire. Model 1 to 10 showing an individual adjustment for each variable. Model 11 was adjusted by all socioeconomic, demographic and health variables (i.e., age, sex, income, immigration status, sick leave, employment type, education, type of injury and diabetes) and presented as adjusted prevalence ration (PR) in Table 2.
